# Anguillulose maligne d’évolution fatale au cours d'un pemphigus végétant

**DOI:** 10.11604/pamj.2015.20.263.6524

**Published:** 2015-03-19

**Authors:** Inssaf Ramli, Hajar Amarouch, Maha Mael-Ainin, Mohammed Aitourhroui, Karima Senouci, Badredine Hassam

**Affiliations:** 1 Service de Dermatologie et Vénéréologie, Faculté de Médecine et de Pharmacie, Université Mohamed V, Centre Hospitalier Universitaire Ibn Sina, avenue Ahmed Balafrej, 10000 Rabat, Maroc

**Keywords:** Albendazole, anguillulose, immunodépression, Ivermectine, Albendazole, anguillulosis, immunodepression, ivermectin

## Abstract

L'anguillulose intestinale est rare. Les formes malignes surviennent généralement au cours d'une immunodépression. Notre observation souligne la gravité de l'anguillulose en cas de maladie auto-immune notamment le pemphigus et l'inefficacité de l'Albendazole dans ces situations à risque.

## Introduction

L'anguillulose est une parasitose fréquente due à Strongylordes stercoralis, prédominant dans la zone intertopicale. Habituellement asymptomatique ou être responsable de troubles digestifs mineurs et, plus rarement, de signes cutanés. L'anguillulose peut, chez les patients immunodéprimés, se généraliser et se compliquer de septicémie et de détresse respiratoire aigue. Nous rapportons un cas original d'une anguillulose maligne associée à un pemphigus végétant chez une jeune patiente candidate d'un traitement immunosuppresseur.

## Patient et observation

**Figure 1:**
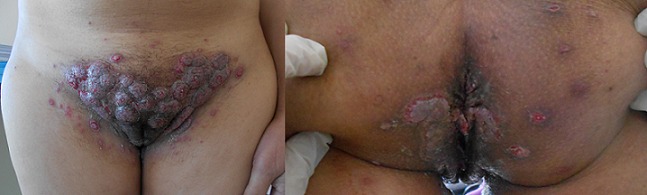
Lésions végétantes de la région pubienne et anale

**Figure 2:**
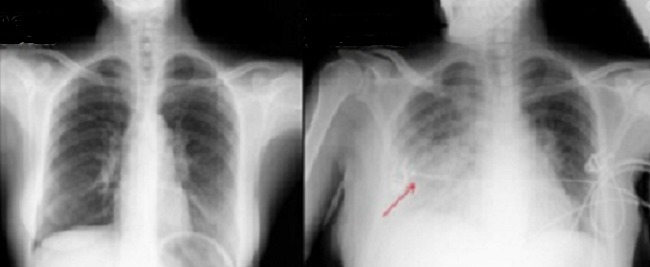
Opacité de l'hémi-champs pulmonaire droit

Mme E. R âgée de 34ans, sans antécédents pathologiques, était hospitalisée pour la prise en charge d'un pemphigus végétant (Figure 1). Le bilan pré-corticothérapie révélait une anguillulose intestinale retenue devant la présence d'une hyperéosinophilie à 3790 éléments/mm^3^ et des Strongyloides Stercoralis à l'examen parasitologique des selles selon la méthode d'extraction de Baermann. Un traitement par Albendazole à la dose de 800 mg/jour était instauré. Au troisième jour du traitement, la patiente présentait brutalement une dyspnée, une hémoptysie et des douleurs basi-thoraciques droites. Elle était fébrile à 39° avec à l′auscultation des râles crépitants à l'hémi-champs pulmonaire droit. Le bilan a montré une anémie régénérative avec une hémoglobine à 7,9g/dl versus 13,2g/dl à l'admission, une opacité occupant la moitié inférieure de l'hémi-champs pulmonaire droit (Figure 2). Devant le terrain de pemphigus, la dyspnée, l'hémoptysie ainsi que l'anémie d'installation brutale et l'opacité pulmonaire, le diagnostic d'une hémorragie pulmonaire survenant dans le cadre d'une anguillulose d'hyperinfestation était retenu. Le traitement par Albendazole était poursuivi vu la non disponibilité de l'Ivermectine dans notre contexte. Une association antibiotique (Teicoplanine et Colimycine) était débutée dans l'attente des résultats des examens bactériologiques et parasitolgiques. Deux jours après, la patiente est décédée suite à une détresse respiratoire aigue en dépit d'une intubation et d'une ventilation mécanique.

## Discussion

L'anguillulose intestinale est Pauci- ou asymptomatique chez le sujet immunocompétent. Elle peut être mortelle en cas d'immunodépression notamment au cours d′une corticothérapie, d'une infection par le VIH ou au cours des maladies auto-immunes [1]. L'anguillulose maligne regroupe deux formes graves: l'anguillulose d'hyperinfestation et l'anguillulose disséminée. Dans la première entité, l'hyperinfestation des viscères est liée à une migration importante des larves strongyloides [2]. L'atteinte pulmonaire peut se manifester par une détresse respiratoire aigue, des pneumonies alvéolaires ou interstielles souvent hémorragiques [2]. L'anguillulose disséminée se caractérise par une défaillance multiviscérale et des septicémies à germes Gram négatifs secondaire au rôle vecteur des parasites [1]. Le traitement de première intention est l'Ivermectine à la dose de 200µg/kg pendant une durée d'au moins trois jours associé à la prise en charge des défaillances viscérales [3]. Dans notre cas, l’évolution était fatale malgré le traitement par Albendazole. La détresse respiratoire aigue serait due à l'hémorragie pulmonaire et/ou à un effet paradoxal du traitement. En effet, la lyse des parasites pourrait entraîner la destruction du parenchyme pulmonaire par réaction inflammatoire intense [4]. Des contrôles coprologiques répétés sont recommandés afin de dépister les rechutes possibles dues à l'existence d'un cycle interne d'auto-infestation du parasite. Certains auteurs proposent un traitement préventif (Thiabendazole 25 mg/kg/j pendant 4 jours) chez les patients atteints d'une maladie chronique nécessitant un traitement corticoïdes ou immunosuppresseur [5].

## Conclusion

L'extension massive d'une anguillulose peut mettre en jeu le pronostic vital. Notre observation souligne la gravité de cette infection en cas de maladie auto-immune notamment le pemphigus, ce qui impose la recherche systématique d'anguillules dans les selles chez tout sujet présentant une cause d'immunodépression.
